# Quetiapine Extended-Release and Peripheral Edema: A Case Report and Literature Review

**DOI:** 10.1155/crps/5806365

**Published:** 2025-10-31

**Authors:** Seshadri Sekhar Chatterjee, Shatavisa Mukherjee, Soumitra Das, Mridula Kayal, Saswata Mondal

**Affiliations:** ^1^Department of Psychiatry, University of Queensland (UQ), Rockhampton, Queensland, Australia; ^2^Department of Psychiatry, Central Queensland Hospital and Health Service (CQHHS), Rockhampton, Queensland, Australia; ^3^Department of Clinical and Experimental Pharmacology, Calcutta School of Tropical Medicine, Kolkata, West Bengal, India; ^4^Department of Psychiatry, Western Health, Melbourne, Victoria, Australia; ^5^Department of Psychiatry, Cairns and Hinterland Hospital and Health Service, Cairns, Queensland, Australia; ^6^Department of Psychiatry, Diamond Harbour Government Medical College and Hospital, Diamond Harbour, West Bengal, India

## Abstract

Quetiapine, an atypical antipsychotic, is widely prescribed for psychiatric disorders, particularly schizophrenia, bipolar disorder, and depressive disorders with or without psychotic symptoms. While edema is more commonly associated with olanzapine and clozapine amongst second general antipsychotics, reports involving quetiapine—particularly the extended-release (XR) formulation—are rare. We describe the case of a 52-year-old woman with severe major depressive episode with psychotic features who was initiated on escitalopram and quetiapine immediate-release (IR) 100 mg/day, later switched to quetiapine XR 100 mg/day to improve adherence and reduce sedation. Ten days after the switch, she developed symmetrical bilateral lower limb pitting edema without systemic symptoms. Comprehensive cardiac, renal, hepatic, thyroid, and immunologic evaluations were unremarkable, and venous Doppler ruled out deep vein thrombosis. The edema resolved within 10 days of discontinuing quetiapine XR and recurred upon rechallenge. The Naranjo Adverse Drug Reaction Probability Score was 9, indicating a probable to definite association. Possible mechanisms include α1-adrenergic blockade, 5-HT2 receptor antagonism, and pharmacokinetic differences in XR formulations leading to sustained peripheral receptor occupancy. Literature review reveals few comparable reports, most involving higher doses or polypharmacy. This case highlights the importance of clinician awareness of quetiapine XR-associated edema, even at low doses, and supports dechallenge–rechallenge as a useful diagnostic approach to improve patient safety and adherence.

## 1. Background

Quetiapine is a second-generation antipsychotic commonly used for various psychiatric indications, including broader off-label use. Its mechanism of action involves antagonism at serotonin receptors (5-HT1A and 5-HT2A), with relatively weak antagonism at dopamine receptors. It also exhibits alpha-adrenergic antagonism, which may explain some of its cardiovascular side effects. Several atypical antipsychotics, such as olanzapine, are known to cause peripheral (especially pedal) edema [1]. However, quetiapine-associated edema is a relatively underexplored topic, with limited literature available. Moreover, the extended-release (XR) formulation has a distinct drug delivery mechanism, potentially contributing to a different side-effect profile. Antipsychotic associated peripheral edema is reported in 1%–10% of patients across various surveillance studies, with higher rates observed for olanzapine and clozapine compared to quetiapine [2]. Pharmacovigilance database analyses, such as the WHO VigiBase and FDA Adverse Event Reporting System (FAERS), have consistently noted edema as an infrequent but clinically relevant adverse event for quetiapine. Despite its low prevalence, the actual incidence may be underestimated due to under-reporting and attribution of edema to other causes, underscoring the importance of case documentation. Here, we report a case of peripheral edema associated with the XRformulation of quetiapine (Qutan SR^R^ 100) and discuss potential mechanisms and supporting literature.

## 2. Case Presentation

### 2.1. Presentation

A 52-year-old woman presented to our psychiatric outpatient clinic with symptoms of persistently low mood, marked lethargy, generalized weakness, anhedonia, and psychotic features including delusions of guilt and reference, along with active suicidal ideation. She had no prior psychiatric history or exposure to psychotropic medications. Based on clinical evaluation, a diagnosis of severe major depressive episode, severe with psychotic features (F32.3) was made.

### 2.2. Medication

The patient was commenced on escitalopram 10 mg daily and quetiapine 25 mg at night, with the quetiapine dose gradually increased to 100 mg per day over 4 days. After 1 month of treatment, her symptoms showed partial improvement, particularly in sleep, appetite, and a reduction in suicidal ideation. To enhance adherence and reduce sedation, the immediate-release (IR) quetiapine was switched to an equivalent dose of quetiapine XR formulation.

However, within 10 days of initiating quetiapine XR, the patient reported gradually progressive bilateral lower limb swelling, particularly around the ankles and shins. The edema was pitting in nature, symmetrical, and not associated with erythema, pain, itching, or systemic symptoms such as fever or dyspnea. There was no recent change in diet, fluid intake, activity level, or use of over-the-counter medications. She had no known history of cardiac, renal, hepatic, or thyroid disease.

### 2.3. Assessment

Physical examination revealed normal blood pressure, pulse rate, respiratory rate, and oxygen saturation. Cardiovascular and respiratory examinations were unremarkable, with no signs of fluid overload or congestive heart failure. Facial or periorbital edema was absent. Comprehensive laboratory investigations were undertaken to determine the etiology of the edema. Hemoglobin, total white blood cell count, and platelet count were within normal limits. Serum electrolytes, including sodium and potassium, were normal. Renal function tests—including blood urea nitrogen and serum creatinine—were unremarkable, and liver function tests, including transaminases and bilirubin, were within normal range. Serum albumin levels were adequate, effectively ruling out hypoalbuminemia as a contributing factor. Thyroid stimulating hormone (TSH) was normal, excluding hypothyroidism. Inflammatory markers such as erythrocyte sedimentation rate (ESR) were not significantly elevated.

Cardiac evaluation with two-dimensional echocardiography revealed a structurally and functionally normal heart, with a preserved left ventricular ejection fraction of 65%. Chest X-ray was normal, showing no signs of cardiomegaly or pulmonary congestion. Immunological testing—including serum IgE and complement components C3 and C4—were within normal limits, making an allergic or autoimmune etiology unlikely. Urinalysis did not reveal proteinuria, hematuria, or other abnormalities. Venous Doppler ultrasound of both lower extremities ruled out deep vein thrombosis.

### 2.4. Management

Given the temporal association with the initiation of quetiapine XR and the exclusion of other medical causes, a provisional diagnosis of quetiapine XR associated peripheral edema was made (Table 1). The quetiapine XR was discontinued and replaced with the IR formulation. Notably, the edema began to resolve within 10 days of this change, without the need for diuretics.

Given the close temporal association between the initiation of quetiapine XR and the onset of symptoms, the resolution upon discontinuation, and the recurrence upon rechallenge, a causal relationship was strongly suspected. The Naranjo Adverse Drug Reaction Probability Score [3] was calculated to be 9, indicating a probable to definite adverse drug reaction to quetiapine XR formulation.

## 3. Discussion

This case highlights a probable adverse effect of peripheral edema associated specifically with quetiapine XR formulation, which resolved on discontinuation and reappeared upon rechallenge. Theoretically, due to their anticholinergic effect and as a part of metabolic side effect, secondary antipsychotics are prone to develop weight gain and edema as well. There are multiple reports of olanzapine being associated with edema and water retention. However, reports with quetiapine are relatively rare in the literature. In our case, the Naranjo scale established that causal and temporal relationship with administration of quetiapine.

### 3.1. Mechanism of Edema

Several potential mechanisms have been proposed to explain quetiapine associated peripheral edema. Alpha-1 adrenergic receptor blockade can lead to vasodilation and increased capillary permeability, facilitating fluid extravasation into the interstitial space. Serotonergic modulation through 5-HT2 receptor antagonism may further influence vascular tone and promote sodium and water retention. Additionally, idiosyncratic hypersensitivity reactions or altered renal sodium handling have been suggested, although these mechanisms were not supported by immunologic or renal investigations in the present case [4, 5].

### 3.2. XR Formulation Consideration

Moreover, as out case reveals, the XR formulation may contribute to a different pharmacokinetic profile, leading to a more sustained plasma concentration and possibly increased exposure at peripheral receptor sites, which could account for the observed reaction. Pharmacokinetic differences between quetiapine IR and XR formulations may contribute to adverse effect variability. The IR formulation typically reaches peak plasma concentration (*T*_max_) within 1.5 h, whereas the XR formulation has a delayed *T*_max_ of approximately 6 h, with reduced Cmax but prolonged drug exposure (higher AUC) [6]. This sustained receptor occupancy may prolong α1-adrenergic and 5-HT2 antagonism, potentially facilitating persistent vascular changes that predispose to edema. Additionally, the XR matrix may alter drug absorption kinetics in a manner that influences peripheral hemodynamics. Additionally, excipients or matrix delivery systems unique to the XR preparation could also play a role, though this remains speculative.

### 3.3. Literature Evidence

On database search, a limited number of case reports have documented quetiapine associated edema [4, 7–10]. Published reports (Table 2) indicate that quetiapine associated peripheral edema typically develops within days to weeks of initiation, most often in patients receiving concomitant agents such as calcium channel blockers or valproate, and resolves rapidly after discontinuation. Onset has been documented at doses ranging from 25 to 800 mg/day, with rechallenge—when attempted—reproducing the reaction. Our patient's presentation, with edema appearing 10 days after switching to the XR formulation, resolution on withdrawal, and recurrence upon rechallenge, mirrors these patterns and highlights the importance of clinician awareness even at lower doses. Several case reports have previously described quetiapine associated edema, with most involving high doses or polypharmacy. However, this case occurred at a relatively low dose (100 mg) and was temporally and reproducibly linked to the XR formulation, underscoring the need for clinician awareness even at lower doses.

### 3.4. Clinical Implication and Conclusion

Clinicians should maintain a high index of suspicion for medication associated edema in patients presenting with new-onset swelling, particularly in the absence of systemic illness. A trial of dechallenge and rechallenge, as performed here, remains a valuable diagnostic tool in such settings.

## Figures and Tables

**Table 1:** Timeline of events.

Timeline event	Day/week	Intervention/clinical event	Outcome
Initiation of quetiapine IR (25 mg nightly, titrated to 100 mg/day)	Day 0	Started with escitalopram 10 mg/day	Improvement in sleep and mood
Switch to quetiapine XR 100 mg/day	Week 4	For better adherence, reduced sedation	—
Onset of bilateral lower limb edema	~Day 10 postswitch	Symmetrical pitting edema ankles/shins	No systemic symptoms
Discontinuation of XR, reintroduction of IR	Immediate	No diuretics given	Gradual resolution over 10 days
Rechallenge with XR	Within 2 weeks	Reappearance of edema	Discontinued XR permanently

**Table 2:** Reported cases.

Study (year)	Age /sex	Indication/comedication/others notes	QTP formulation and dose	Time to onset	Time to resolution	Rechallenge
Rozzini et al. [7]	72/M	Lewy body dementia; CCB long-term	IR ↑ to 150 mg/day (50 mg AM + 100 mg PM)	1 day after dose increase	Improved after discontinuation (exact days NR)	Not attempted
Koleva et al. [4]	59/F; 44/F; 38/F	Mixed psychiatric; several on meds incl. CCB/NSAIDs	150–500 mg; 50 mg; 200 mg	Day 8; 2 days; 1 day	NR; 2 days; ~1 week	One patient had prior edema on olanzapine; improvement on dose ↓ in one case
Chen et al. [8]	73/F	BPAD I; long-term valproate 1000 mg/day	IR 100 mg/day added to valproate	Day 7	5 days after stop	Not attempted
Polat et al. [9]	46/F; 52/F; 43/F; 50/F; 22/F	Bipolar spectrum; several on valproate	200–600; 200–400; 300–600; 800; 300–600 mg	2 weeks; 2 weeks; 2 weeks; 5 days; unclear	7 days; 10 days; 3 days; 7 days; 7 days	Not reported
Geldenhuys et al. [10]	38/M	BPAD I; valproate 700 mg BID; zuclopenthixol depot	200 mg BID (400 mg/day)	After ~3 months on QTP (≈2 months postdischarge)	3 days after stopping	Not attempted
Tuman et al. [11]	26/F	BPAD; caused angioedema	400 mg daily	1 day after dose increase	2 days after stopping	Not attempted
Elyasi et al. [12]	20/F	Delirium and chest trauma; caused chest wall edema	Started at 25 mg and increased upto 225 mg daily	6 days after starting	Completely subsided 2 days after stopping	Not reported
Roy et al. [5]	43/F	Depressive disorder and PTSD, peripheral edema; sertraline, buprenorphine	300 mg	Not mentioned	Subsided after stopping, time not mentioned	Not attempted

## Data Availability

The data that support the findings of this study are available from the corresponding author upon reasonable request.

## References

[1] Dahal S., Banjade R. R., Kafle P. K. (2025). Olanzapine-Induced Ankle Edema: A Case Report on a Rare Adverse Effect. *Psychiatry Research Case Reports*.

[2] Engel J., Haack B., Zolk O. (2024). Edema Related to Treatment With Psychotropic Drugs. *Journal of Neural Transmission*.

[3] Naranjo C. A., Busto U., Sellers E. M. (1981). A Method for Estimating the Probability of Adverse Drug Reactions. *Clinical Pharmacology and Therapeutics*.

[4] Koleva H. K., Erickson M. A., Vanderlip E. R., Tansey J., Mac J., Fiedorowicz J. G. (2009). 3 Case reports of Edema Associated With Quetiapine. *Annals of Clinical Psychiatry*.

[5] Roy K., Astreika V., Dunn J. E., Sappati Biyyani R. S. R. (2009). Quetiapine-Induced Peripheral Edema. *General Hospital Psychiatry*.

[6] Joshi K., Rao S., Mehta S. (2025). A Review of Pharmacokinetic and Pharmacodynamic Properties of Quetiapine IR and XR: Insights and Clinical Practice Implications. *Cureus*.

[7] Rozzini L., Ghianda D., Vicini Chilovi B., Padovani A., Trabucchi M. (2005). Peripheral Oedema Related to Quetiapine Therapy. *Drugs and Aging*.

[8] Chen C. Y., Yeh Y. W., Kuo S. C., Shiah I. S., Liu P. Y., Chen C. L. (2009). Pedal Edema Associated With Addition of Low-Dose Quetiapine to Valproate Treatment in Bipolar Disorder. *Progress in Neuro-Psychopharmacology and Biological Psychiatry*.

[9] Polat A., Turan H., Gok R., Gunduz N. (2019). Quetiapıne-Induced Peripheral Edema: Case Series. *Dusunen Adam: The Journal of Psychiatry and Neurological Sciences*.

[10] Geldenhuys C., Payne Maziena A., Steyn P. (2024). Quetiapine-Induced Peripheral Oedema: A Case Report and Review of the Literature. *Personalized Medicine in Psychiatry*.

[11] Tuman T. C., Tuman B. A., Sereflican B., Yildirim O. (2016). Quetiapine Associated With Angioedema. *Journal of Clinical Psychopharmacology*.

[12] Elyasi F., Heydari F., Haddadi K. (2023). Quetiapine-Induced Chest Wall Edema With the Swellings of Face and Extremities in a Young Hospitalized Patient: A Case Report. *Clinical Case Reports*.

